# Multimodality locoregional treatment strategies for bridging HCC patients before liver transplantation

**DOI:** 10.1007/s10353-017-0487-8

**Published:** 2017-09-04

**Authors:** Georg P. Györi, D. Moritz Felsenreich, Gerd R. Silberhumer, Thomas Soliman, Gabriela A. Berlakovich

**Affiliations:** Department of Surgery, Division of Transplantation, Medical University of Vienna, Währinger Gürtel 18–20, 1090 Vienna, Austria

**Keywords:** Hepatocellular carcinoma, Liver transplantation, Ablation techniques, Bridging, Downstaging

## Abstract

**Background:**

It is current practice that patients with hepatocellular carcinoma (HCC) listed for liver transplantation should receive locoregional treatment if the suspected waiting time for transplantation is longer than 6 months, even in the absence of prospective randomized data. Aim of this study was the comparison of single versus multimodality locoregional treatment strategies on outcomes after liver transplantation.

**Methods:**

This is a retrospective analysis of 150 HCC patients listed for liver transplantation at our center between 2004 and 2011. Outcomes were analyzed according to modified Response Evaluation Criteria in Solid Tumors (mRECIST) in relation to intention-to-treat and overall survival after liver transplantation.

**Results:**

Overall, 92 patients (63%) were transplanted in this cohort. The intention-to-treat 1‑, 3‑, 5‑year waiting list survival was 80, 59, and 50% respectively. In RFA-(radiofrequency ablative) and TACE-(transarterial chemoembolisation)-based regimens, rates of transplanted patients were comparable (69 vs. 58%, *p* = ns). No difference was seen in overall survival after liver transplantation when comparing TACE- and RFA-based regimens. Patients receiving multimodality locoregional therapy had lower overall survival after transplantation (*p* = 0.05)

**Conclusion:**

TACE- and RFA-based regimens showed equal outcomes in terms of transplantation rate, tumor response, and post-transplant survival. Patients in need of more than one treatment modality might identify a cohort with poorer post-transplant survival.

**Points of novelty:**

Direct comparison of TACE and RFA in a multimodality setting, analysis according to mRECIST.

## Introduction

Orthotropic liver transplantation (OLT) is the standard curative treatment for selected patients with hepatocellular carcinoma (HCC), considering that the majority present with concomitant cirrhosis at the time of diagnosis and are not amenable to resection [1].

Restrictions in tumor size and number of nodules have been implemented in order to establish good post-transplantation outcomes [2, 3]. Over the past decade, many centers were able to extend the selection criteria while maintaining comparable outcomes [4, 5]. Even though HCC patients within certain selection criteria are prioritized in terms of allocation throughout the world, tumor progression and waiting list dropouts represent significant problems in the management of HCC patients [6, 7].

Locoregional therapies (LRT) deliver toxic thermal/chemical/radioactive doses to tumors with minimal toxicity to normal tissue. Transarterial chemoembolization (TACE) and yttrium-90 radioembolization are LRTs that have demonstrated a palliative role in HCC patients [8]. Their role in downstaging transplant patients and bridging patients to transplantation is currently under debate, and data are scarce. Locoregional therapies have been successfully used to prevent tumor progression on the waiting list [9].

For HCC lesions under 3 cm (single or up to three), radiofrequency ablation (RFA) is the preferred LRT; for multinodular tumors (more than three lesions) or single lesions more than 3 cm, TACE is the preferred LRT [10].

Additionally, patients primarily beyond listing criteria were added to the waiting lists after tumor reduction via LRTs [11–13].

Aim of this study was the comparison of locoregional treatment strategies on both waiting list and transplant survival in a large patient series.

## Patients and methods

All HCC patients listed for liver transplantation in our center between January 2004 and December 2011 were included in this retrospective analysis.

Collected data included standard demographic data (age, gender) as well as preoperative staging, Milan criteria status, date of listing, waiting time, modality of locoregional therapy, number of treatments, and treatment-associated morbidity including severe adverse events (SAE) within 4 weeks.

An intention-to-treat (ITT) analysis was performed for all patients. In addition, patients were grouped according to their main pre-transplant ablative regimes used for bridging to transplantation: (i) transarterial chemoembolization, (ii) ablative strategies such as radiofrequency ablation in combination with/without percutaneous alcohol instillation (PEI), or (iii) no bridging. For patients receiving more than one LRT modality, subgroup analysis was performed (mmLRT).

Routine CT scans for tumor evaluation were performed at the time of listing, as well as every 3 months during the waiting time.

Histological examination of all explanted livers was performed in all transplanted patients.

Response to LRT was grouped as described in the modified Response Evaluation Criteria in Solid Tumors (mRECIST): complete response: CR (tumor necrosis 100%); partial response: PR (decrease in the sum of diameters 30%); stable disease: SD (no partial response or no progressive disease); or progressive disease: PD (20% increase in the sum of the diameters) [10].

Routine follow-up consisted of clinical and radiological examination every 6 months after transplantation.

Outcome parameters were percentage of patients reaching transplantation, downstaging, tumor necrosis rate, overall survival after listing, and overall survival after liver transplantation.

### Statistical analysis

Continuous data are given as median and interquartile range (IQR; range from the 25^th^ to the 75^th^ percentile), or mean and standard deviation, where appropriate. Discrete data are presented as counts and percentages. Chi-square tests or, if appropriate, exact tests were used to compare groups of categorical data. For comparisons of continuous data a Mann-Whitney U test was performed. Kaplan–Meier survival estimates were used to calculate graft and patient survival, and the Mantel–Cox log-rank test was used to compare survival between groups. A two-sided *p*-value of <0.05 was considered statistically significant. All calculations were performed using SPSS for Mac 19 (IBM SPSS Statistics for Macintosh, Version 19, Armonk, NW: IBM Corp.).

The study was approved by the Medical University of Vienna Ethics Board (Research ethics reference number 0994/2010).

## Results

**Fig. 1:**
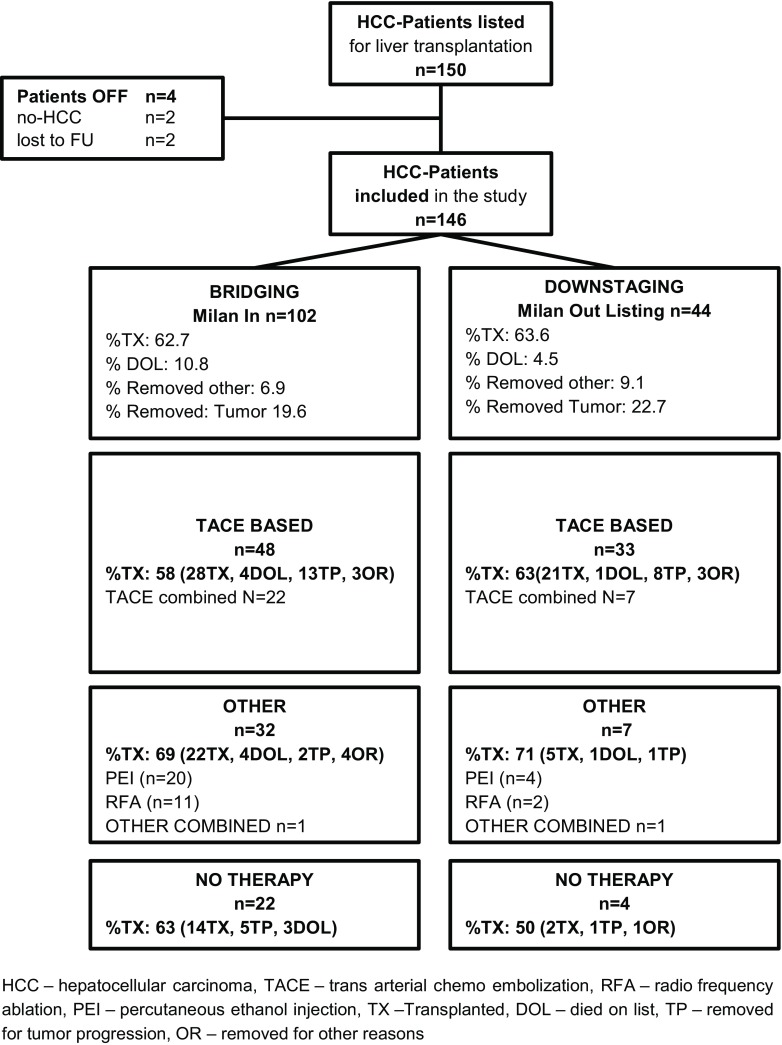
Study selection flowchart of HCC patients receiving liver transplantation. *HCC* hepatocellular carcinoma, *TACE* transarterial chemoembolization, *RFA* radiofrequency ablation, *PEI* percutaneous ethanol injection, *TX* transplanted, *DOL* died on list, *TP* removed for tumor progression, *OR* removed for other reasons, *FU* follow-up

We identified 150 patients with hepatocellular carcinoma matching the primary study criteria. A total of 4 patients had to be excluded from analysis for removal from the waiting list for non-HCC-related reasons, leaving a total of 146 patients for final analysis. The patient selection flowchart is depicted in Fig. 1.

The mean age was 56.9 (±7.6) years. 132 (86%) patients were male, 20 (14%) were female. 63 (43%) patients had hepatitis C, 43 (29%) had alcoholic cirrhosis, 11 (8%) had hepatitis B, and 30 (20%) patients hat combined or other etiologies of liver cirrhosis. Baseline demographic data are provided in Table 1.

**Table 1:** Patient characteristics and overall survival data for all patients listed for liver transplantation (*N* = 146)

	All patients *N* = 146	Milan in listing *N* = 102			*p*-value	Milan out listing *N* = 44			*p*-value
		TACE + *N* = 48	PEI/RFA *N* = 32	NO *N* = 22		TACE + *N* = 33	PEI/RFA N = 7	NO *N* = 4	
*Mean age (years)*	**56.9 ±** **7.6**	56.9 ± 8.2	56.2 ± 7.5	55.2 ± 7.3	*0.72*	59.1 ± 5.9	58.0 ± 6.7	52.3 ± 5.0	*0.11*
*Sex (% female)*	**14**	17	12	23	*0.62*	9	14	0	*0.75*
*Mean nr. treatments*	**1.8 ±** **1.6**	2.6 ± 1.7	1.9 ± 1.3	–	*0.012*	2.2 ± 1.6	1.7 ± 1.1	–	*0.44*
*Mean WT (months)*	**8.2 ±** **5.5**	9.1 ± 5.7	8.9 ± 5.0	7.0 ± 5.8	*0.32*	7.8 ± 5.9	5.3 ± 2.5	5.3 ± 4.5	*0.42*
*% transplanted*	**63**	58	69	64	*0.65*	64	71	50	*0.79*
*Cirrhosis etiology*	–	–	–	–	*0.049*	–	–	–	*0.62*
PHCC	**62**	13	18	10	–	15	3	3	–
ALCI	**43**	16	9	9	–	8	1	0	–
PHBC	**11**	6	0	0	–	4	1	0	–
Other	**16**	7	2	1	–	6	0	0	–
Combined	**14**	6	3	2	–	0	2	1	–
*Mean nr. of nodules*	**2.3 ±** **1.9**	1.5 ± 0.8	1.4 ± 0.8	1.5 ± 0.70	*0.34*	3.9 ± 2.0	5.1 ± 2.2	6.3 ± 2.4	*0.066*
*Mean size of nodules*	**2.5 ±** **1.0**	2.6 ± 1.2	2.5 ± 0.9	2.2 ± 1.1	*0.14*	2.2 ± 1.0	2.2 ± 0.8	1.6 ± 0.6	*0.48*
*Survival listing %*	–	–	–	–	*0.37*	–	–	–	*0.50*
1 year	**80%**	80%	80%	78%	–	80%	72%	100%	–
3 year	**59%**	41%	66%	60%	–	50%	58%	75%	–
5 year	**50%**	41%	60%	48%	–	50%	30%	38%	–
MOS	**33.4 ±** **26.9**	29.1 ± 21.9	34.7 ± 30.4	44.1 ± 31.1	–	32.3 ± 24.8	33.1 ± 38.8	49.6 ± 15.6	–

*ALCI* alcoholic cirrhosis, *NO* no treatment, *PEI* percutaneous ethanol instillation, *PHCC* post hepatitis C cirrhosis, *PHBC* post hpeatits B cirrhosis, *RFA* radiofrequency ablation, *TACE* transarterial chemoembolisation, *WT* waiting time

63% (92 patients) of this cohort underwent OLT, 21% (30 patients) were removed due to tumor progression, 9% (13 patients) died while waiting for transplant. The mean time on the waiting list was 8.2 (±5.5) months. Mean tumor size was 2.5 (±1.1) cm, mean number of nodules was 2.3 (±1.9).

At time of listing, 70% (102 patients) were within Milan criteria (MC_IN), whereas 30% (44 patients) were beyond Milan criteria (MC_OUT).

### Locoregional therapy (LRT)

In this cohort, 55% (81 patients) received TACE-based locoregional therapy, 27% (39 patients) a PEI/RFA regimen, and 18% (26 patients) had no treatment while on the waiting list. Detailed information is provided in Fig. 1.

Overall, a mean of 1.8 (±1.6) sessions were performed. Treatments performed were not significantly different between groups.

### Multimodality locoregional therapy

In 29 patients a combined—multimodality—LRT (mmLRT) was performed: TACE and PEI in 14 (48%), TACE and RFA in 11 (38%), and PEI and RFA in 2 (7%), and TACE/PEI/RFA in 2 (7%).

There was a trend towards longer waiting time in patients receiving mmLRT (10.2 vs. 8 months, *p* = 0.07). Lesion size or numbers were not significantly different between groups. Detailed information on LRT is shown in Fig. 1.

### Endpoint transplantation

Overall, 92 patients (63%) were transplanted. Transplant rates were not different for patients within and beyond Milan criteria, 63% vs. 63% respectively. TACE- and RFA/PEI-based LRT also showed equal transplant rates; detailed data is provided in Fig. 1.

### Response according to LRT

Using mRECIST, overall, 25 (27.2%) patients showed a complete response (CR), 29 (31.5%) patients showed a partial response (PR), 26 patients (28.3%) had stable disease (SD), and 12 patients (13.0%) had progressive disease (PD) to/during LRT.

Patients receiving TACE had the highest CR rate (41.2% vs. PEI 30.0%, RFA 33.3%). Patients receiving combined treatments mmLRT showed the lowest rate of CR (12.5%).

Furthermore, patients receiving RFA also showed the highest necrosis rate. (84.9% vs. TACE 72.0% and PEI 64.9%). Patients receiving mmLRT showed the lowest necrosis rate 56.4% (*p* = 0.15).

### Downstaging

At the time of listing, 44 patients were beyond Milan criteria (MC_OUT). Eleven patients (25%) who were MC_OUT at time of listing were not transplanted.

Nineteen patients were successfully downstaged to MC_IN: TACE was used in 13 patients (46.4%), PEI in 3 patients (10.7%), RFA in 1 pt (3.6%), and 2 patients (7.1%) received mmLRT. Downstaging was verified in explanted liver specimens. Detailed information is seen in Table 2 **.**

**Table 2:** Tumor characteristics, mRECIST, and survival in patients after liver transplantation (*N* = 92)

	All patients *N* = 92	Milan in listing *N* = 64			*P*-value	Milan out listing *N* = 28			*P*-value
		TACE + *N* = 28	PEI/RFA *N* = 22	NO *N* = 14		TACE + *N* = 21	PEI/RFA *N* = 5	NO *N* = 2	
*Grading G*	–	–	–	–	*0.13*	–	–	–	*0.14*
G0	**13**	2	6	3	–	2	0	0	–
G1	**2**	0	0	1	–	1	0	0	–
G2	**75**	26	16	10	–	18	4	1	–
G3	**2**	0	0	0	–	0	1	1	–
*Staging T*	–	–	–	–	*0.19*	–	–	–	*0.97*
T0	**13**	2	6	3	–	2	0	0	–
T1	**27**	10	6	3	–	5	2	1	–
T2	**44**	11	9	7	–	13	3	1	–
T3	**7**	4	1	1	–	1	0	0	–
T4	**1**	1	0	0	–	0	0	0	–
*Staging N*	–	–	–	–	–	–	–	–	–
N0	**92**	28	22	14	–	21	5	2	–
N1	**0**	0	0	0	–	0	0	0	–
*Staging V*	–	–	–	–	*0.22*	–	–	–	*0.58*
V0	**68**	18	18	12	–	14	4	2	–
V1	**24**	10	4	2	–	7	1	0	–
*% necrosis*	**68% ±** **36**	58% ± 39	72% ± 35	–	*0.18*	80% ± 24	56% ± 43	–	*0.11*
*Survival TX % N = 92*	–	–	–	–	*0.17*	–	–	–	*0.76*
1 year	**82%**	93%	79%	85%	–	80%	60%	100%	–
3 year	**78%**	83%	65%	75%	–	80%	60%	100%	–
5 year	**76%**	65%	65%	75%	–	80%	30%	100%	–
Mean OS	**35.3 ±** **28.1**	29.7 ± 25.6	36.3 ± 30.1	38.6 ± 32.1	–	32.6 ± 27.5	36.4 ± 41.4	48.9 ± 20.6	–
*mRECIST*	–	–	–	–	*<0.005*	–	–	–	*0.091*
CR	**25**	7	6	–	–	9	2	–	–
PR	**29**	7	11	–	–	7	2	–	–
SD	**26**	9	5	10	–	3	1	1	–
PD	**12**	5	–	4	–	2	–	1	–

*NO* no treatment, *PEI* percutaneous ethanol instillation, *RFA* radiofrequency ablation, *TACE* transarterial chemoembolisation

### Severe adverse events

In this cohort, a total of 12 severe adverse events (SAE) were recorded, 6 patients died, 2 patients were delisted. 4 patients were transplanted.

Detailed information is provided in Table 3. No significant difference between TACE and other LRTs was seen.

**Table 3:** Severe adverse events 4 weeks after LRT

Portal vein thrombosis	TACE	Died on list
Portal vein thrombosis	TACE	Died on list
Portal vein thrombosis	TACE	Transplanted
Portal vein thrombosis	TACE	Transplanted
Portal vein thrombosis	PEI	Transplanted
Ascites	RFA	OFF list
Insult	RFA	OFF list
Caput pancreas necrosis	TACE	Transplanted
Caput pancreas necrosis	TACE	Died on list
Other	TACE	Died on list
Other	PEI	Died on list
Other	TACE	Transplanted

*PEI* percutaneous ethanol instillation, *RFA* radiofrequency ablation, *TACE* transarterial chemoembolisation

### Survival

#### Intention-to-treat survival analysis

**Fig. 2:**
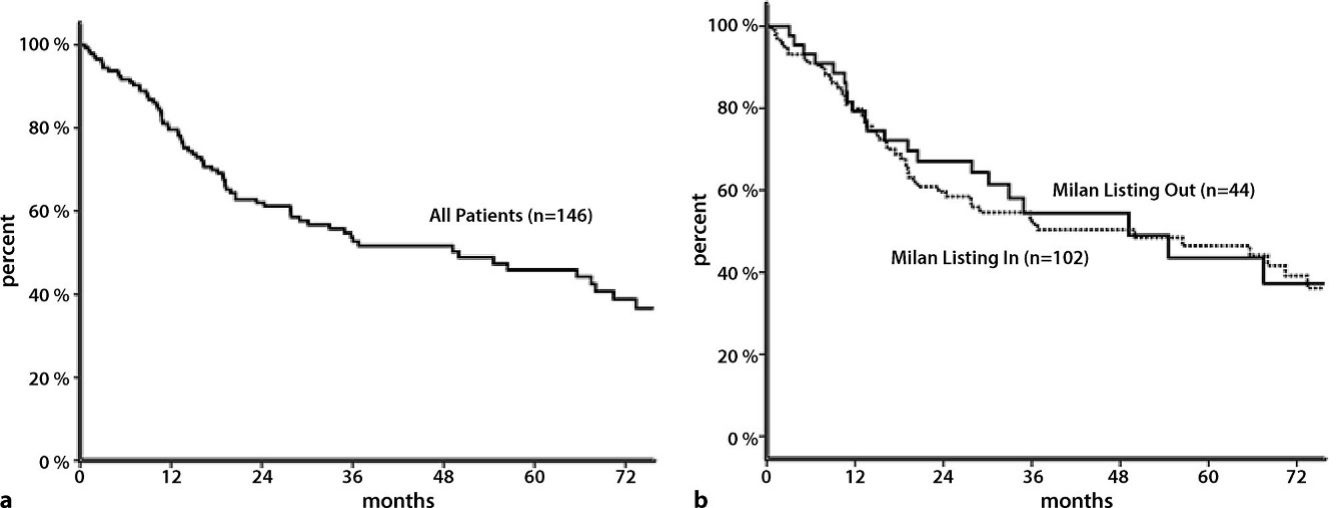
**a** Kaplan–Meier survival: intention-to-treat survival from listing (*N* = 146). **b** Kaplan–Meier survival: intention-to-treat survival from listing according to Milan criteria (*N* = 146)

One-, 3‑, and 5‑year ITT waiting list survival was 80%, 59%, and 50% respectively (Fig. 2a). No difference was seen in ITT survival for patients within or beyond Milan criteria (Fig. 2b).

Overall survival from listing was comparable between LRT treatment groups (Table 1).

#### Post-transplant survival

**Fig. 3:**
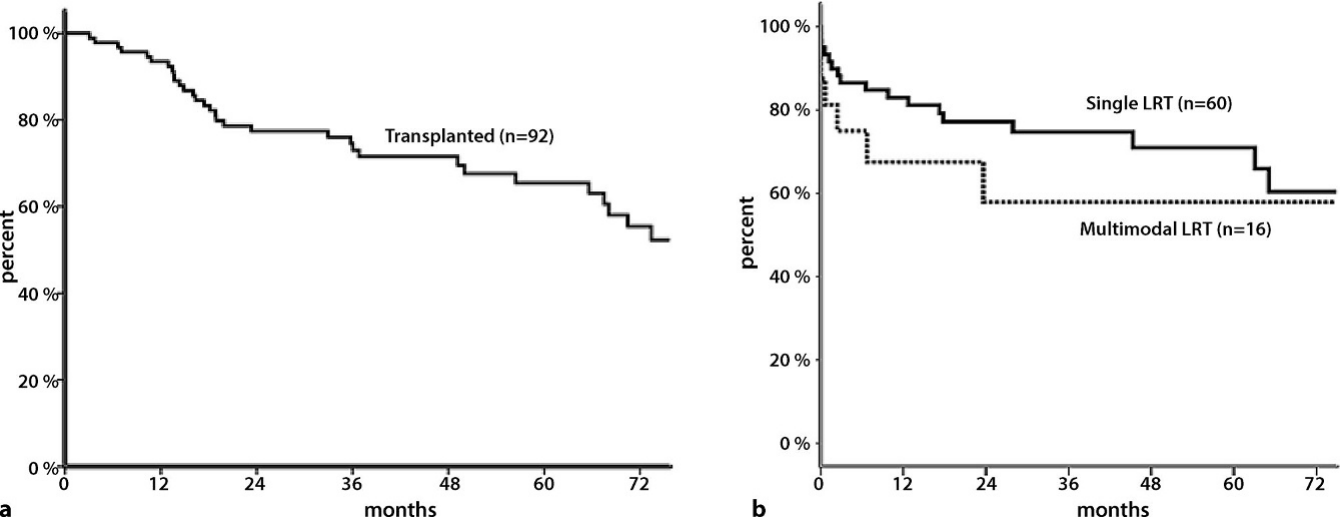
**a** Kaplan–Meier survival: overall post-transplant survival (*N* = 92). **b** Kaplan–Meier survival: overall post-transplant survival according to single or multimodality locoregional treatment (LRT) (*N* = 76)

Overall 1‑, 3‑, and 5‑year post-transplant survival of 92 transplanted patients was 82%, 78%, and 76% respectively (Fig. 3a).

One-, 3‑, and 5‑year overall survival were comparable for downstaged and non-downstaged patients at 85% vs. 94%, 74% vs. 72%, and 67% vs. 68% respectively (*p* = 0.67).

Patients who received multimodal LRT (mmLRT) showed a lower 1‑, 3‑, and 5‑year post-transplant survival when compared with patients who received any single LRT (68%, 58%, and 58% versus 82%, 75%, and 70%; *p* = 0.05; Fig. 3b).

## Discussion

This study evaluates different locoregional treatment strategies for hepatocellular carcinoma patients listed for transplantation. We found that patients who are in need of multiple types of treatment showed somewhat lower overall survival after transplantation.

A variety of publications exist on locoregional therapies for HCC before transplantation. Even though there is a lack of prospective RCTs, there is consensus that patients with HCC and an expected waiting time of longer than 6 months should undergo locoregional therapy for HCC [14]. To this day, no single strategy has proved to be superior in terms of tumor response, dropout from the waiting list, and outcome after transplantation [15]. TACE has reported tumor response rates up to almost 60% [16–21], and RFA is reported to be somewhat higher [22–24]. Our data are in concordance with these previous findings, showing a mean necrosis rate of 58% for TACE and 72% for RFA, not reaching significant difference. Data on waiting list dropouts are limited in previous reports, especially for cohorts with RFA [15]. Dropout rates vary highly in reports between 3 and 35%, for TACE and up to 25% for RFA [17, 19, 22, 23, 25, 26]. These reports should be interpreted with caution, as waiting times differ significantly and some are pre-MELD era publications. This cohort of patients who underwent bridging or downstaging have comparable transplant rates. In addition, direct comparison between different LRT strategies showed no significant difference in dropout rates between groups. It is noteworthy that severe adverse events were evenly distributed and did not negatively impact the transplantation rate.

Previous studies report the 5‑year overall post-transplant survival for HCC to be around 65% [15, 18]. Patients with tumors that have complete necrosis after TACE might have beneficial outcome [19]; a clear survival benefit for patients with any LRT has not been proven so far [15, 27, 28]. We found equal intention-to-treat and post-transplant survival rates for all patients in this cohort. Patients receiving more than one type of bridging therapy, however, showed a somewhat lower post-transplant survival. Tumor size was not different between groups. Thus this fact might be indicative of poorer tumor biology. Data on multimodality treatment for HCC in the transplant setting are limited to the setting of unresectable HCC larger than 3 cm [29, 30]. Only one study evaluating multimodality treatment in 44 patients with early stage HCC was identified, reporting 76% mean necrosis and a low transplant rate of 44% [31].

We are aware that this study has some limitations. First of all, it is retrospective in design. In addition, despite the large cohort size, the treatment groups are unevenly distributed, with patients receiving TACE being the largest group. However, no significant differences in tumor-related baseline parameters (size of nodules, number of nodules) or baseline demographic data were seen between groups.

In conclusion, our data show that patients with or without locoregional therapies have comparable long-term survival when transplanted. TACE and ablative strategies such as RFA are equally effective in bridging to transplantation and downstaging. Patients in need of more than one treatment modality might identify a cohort with inferior post-transplant survival.
